# Annual trading patterns and risk factors of avian influenza A/H5 and A/H9 virus circulation in turkey birds (*Meleagris gallopavo*) at live bird markets in Dhaka city, Bangladesh

**DOI:** 10.3389/fvets.2023.1148615

**Published:** 2023-07-04

**Authors:** Ariful Islam, Emama Amin, Shariful Islam, Mohammad Enayet Hossain, Abdullah Al Mamun, Md. Sahabuddin, Mohammed Abdus Samad, Tahmina Shirin, Mohammed Ziaur Rahman, Mohammad Mahmudul Hassan

**Affiliations:** ^1^EcoHealth Alliance, New York, NY, United States; ^2^Centre for Integrative Ecology, School of Life and Environmental Sciences, Deakin University, Geelong, VIC, Australia; ^3^Institute of Epidemiology, Disease Control and Research (IEDCR), Dhaka, Bangladesh; ^4^One Health Laboratory, International Centre for Diarrheal Diseases Research, Bangladesh (icddr,b), Dhaka, Bangladesh; ^5^National Reference Laboratory for Avian Influenza, Bangladesh Livestock Research Institute (BLRI), Savar, Bangladesh; ^6^Queensland Alliance for One Health Sciences, School of Veterinary Science, The University of Queensland, Brisbane, QLD, Australia; ^7^Faculty of Veterinary Medicine, Chattogram Veterinary and Animal Sciences University, Chattogram, Bangladesh

**Keywords:** surveillance, avian influenza, LBM, trading, economics

## Abstract

The impacts of the avian influenza virus (AIV) on farmed poultry and wild birds affect human health, livelihoods, food security, and international trade. The movement patterns of turkey birds from farms to live bird markets (LBMs) and infection of AIV are poorly understood in Bangladesh. Thus, we conducted weekly longitudinal surveillance in LBMs to understand the trading patterns, temporal trends, and risk factors of AIV circulation in turkey birds. We sampled a total of 423 turkeys from two LBMs in Dhaka between May 2018 and September 2019. We tested the swab samples for the AIV matrix gene (M-gene) followed by H5, H7, and H9 subtypes using real-time reverse transcriptase-polymerase chain reaction (rRT-PCR). We used exploratory analysis to investigate trading patterns, annual cyclic trends of AIV and its subtypes, and a generalized estimating equation (GEE) logistic model to determine the factors that influence the infection of H5 and H9 in turkeys. Furthermore, we conducted an observational study and informal interviews with traders and vendors to record turkey trading patterns, demand, and supply and turkey handling practices in LBM. We found that all trade routes of turkey birds to northern Dhaka are unidirectional and originate from the northwestern and southern regions of Bangladesh. The number of trades from the source district to Dhaka depends on the turkey density. The median distance that turkey was traded from its source district to Dhaka was 188 km (Q1 = 165, Q3 = 210, IQR = 45.5). We observed seasonal variation in the median and average distance of turkey. The qualitative findings revealed that turkey farming initially became reasonably profitable in 2018 and at the beginning of 2019. However, the fall in demand and production in the middle of 2019 may be related to unstable market pricing, high feed costs, a shortfall of adequate marketing facilities, poor consumer knowledge, and a lack of advertising. The overall prevalence of AIV, H5, and H9 subtypes in turkeys was 31% (95% CI: 26.6–35.4), 16.3% (95% CI: 12.8–19.8), and 10.2% (95% CI: 7.3–13.1) respectively. None of the samples were positive for H7. The circulation of AIV and H9 across the annual cycle showed no seasonality, whereas the circulation of H5 showed significant seasonality. The GEE revealed that detection of AIV increases in retail vendor business (OR: 1.71; 95% CI: 1.12–2.62) and the bird’s health status is sick (OR: 10.77; 95% CI: 4.31–26.94) or dead (OR: 11.33; 95% CI: 4.30–29.89). We also observed that winter season (OR: 5.83; 95% CI: 2.80–12.14) than summer season, dead birds (OR: 61.71; 95% CI: 25.78–147.75) and sick birds (OR 8.33; 95% CI: 3.36–20.64) compared to healthy birds has a higher risk of H5 infection in turkeys. This study revealed that the turkeys movements vary by time and season from the farm to the LBM. This surveillance indicated year-round circulation of AIV with H5 and H9 subtypes in turkey birds in LBMs. The seasonality and health condition of birds influence H5 infection in birds. The trading pattern of turkey may play a role in the transmission of AIV viruses in the birds. The selling of sick turkeys infected with H5 and H9 highlights the possibility of virus transmission to other species of birds sold at LBMs and to people.

## 1. Introduction

Turkey (*Meleagris gallopavo*) is a large gallinaceous bird of the family Meleagridae. In addition to chicken, duck, guinea fowl, and quail, turkey maintains a significant position and substantially improves the nutritional and economic conditions of diverse people worldwide (1). They make up around 1.2% of the total poultry population of the world (2). Turkeys are grown primarily for their premium and exquisite meat across the world. Turkey meat is the second largest contributor to the world’s poultry meat production after chicken (3). Turkeys are vital in providing animal protein to the Western world, mainly Europe and America. The birds are raised specifically for their meat production. Because turkey meat is among the leanest of any domestic poultry species due to its unique flavor, texture, and quality (4). According to the turkey management guide 2012, turkey has a high dressing percentage that could amount to 87% of the slaughter weight (5).

Poultry meat supplies 37% of Bangladesh’s domestic meat requirements (6). Turkey is a relatively new poultry species in Bangladesh, and this species’ meat is most likely one of the most significant alternatives to protein sources in Bangladesh (4). Turkey’s production is a prominent and highly profitable agricultural industry with growing worldwide demand for its products (7). Since turkey has recently gained popularity in Bangladesh, farmers are inexperienced with several aspects of rearing, such as feeding, housing, disease prevention and management, standard growth pattern, feed efficiency, and hatching egg incubation. Moreover, turkeys are more susceptible to avian influenza virus (AIV) infection than other poultry species (8, 9). In several countries, both highly pathogenic avian influenza (HPAI) and low pathogenic avian influenza (LPAI) subtypes have been detected in turkeys (10–12). In 2017 H5N1 infections were detected in turkey farms in Bangladesh (13, 14).

The AIV has received increasing attention over the years due to its impact on production, trade, and human health and causes severe economic losses when an outbreak occurs in chickens, turkeys, and other gallinaceous birds (15–17). The H5N1 virus has become prevalent in the poultry populations of Southeast Asian countries, including Bangladesh, and has resulted in substantial economic loss, human illness, and death (18, 19). Bangladesh reported 585 HPAI H5N1 outbreaks in poultry and wild bird from 2007 to 2022, with 90% of those cases coming from commercial poultry farms (20, 21). Since 2008, Bangladesh has recorded three human cases of H9N2 and eight H5N1 cases, including one death; 3 of these cases were among LBM workers who were likely exposed to infected poultry there (20, 22, 23).

Bangladesh is one of the most densely populated countries, in the world, with human 160 million and 1,072 people/km^2^, and is considered a low to middle-income country in South Asia (24) with an agriculture and livestock-based economy (25). Bangladesh’s estimated poultry population is about 258.23 million, comprising 189.26 million chickens, 67.52 million domestic ducks, and 1.44 million domestic turkeys (26). In combination with an intensifying farming system and substantial poultry population (1,194 birds/km^2^) (27), make Bangladesh a significant candidate for being the source of newly emerging influenza strains with pandemic-causing potential. Multiple clades of H5N1, including 2.2.2, 2.3.2, 2.3.4.2 (28, 29), 2.3.2.1a (30, 31), and 2.3.4.4b (32) clade of H5N6 have been found in both poultry and wild birds in Bangladesh since the initial discovery of viruses. A new reassortant clade 2.3.2.1a of H5N1 was detected from multiple clinical outbreaks in ducks, geese, chickens, crows, and turkeys in Bangladesh (13, 15, 30).

On the other hand, Live bird markets (LBMs) have been recognized as critical habitats for the persistence, propagation, replication, and spread of AIV over the years (22, 33). The first evidence of zoonotic influenza H5N1 transmission in Bangladesh was in 2012; LBMs in Dhaka were the primary infection source for all three human cases. Multiple studies have detected HPAI and LPAI in different poultry species and environmental surfaces in LBMs (20, 34, 35). Bangladesh has many LBMs in urban, peri-urban, and rural areas where multiple poultry species from the backyard and commercial farms are kept together for sale (36, 37). In Bangladesh, LBMs serve as the country’s principal marketing hub for poultry and the consistent supply of live birds and thus act as potential centers for pathogen accumulation, amplification, and ongoing spread of various infectious diseases, including AIV (37–39). LBMs are typically at the center of an intricate trading network that brings together a wide diversity of bird species typically produced by several farmers in different regions of the country utilizing a wide range of poultry production practices (40). The different poultry species are frequently marketed alive at LBMs due to cultural and religious traditions for eating freshly slain birds and the absence of refrigeration, particularly in village areas (22). The type of poultry raised in Bangladesh significantly correlates with the husbandry practices used and the geographic location of the poultry farms. Hence, we predicted that trading patterns of live poultry might vary depending on the species of fowl (41).

The trading networks of turkey birds in Bangladesh are poorly or entirely undocumented. Due to a lack of understanding of this trading system, it is challenging to implement adequate and targeted AIV prevention measures. This surveillance was conducted to address the dearth of information regarding turkey bird trading routes and the potential impact of an AIV outbreak on such a system and to investigate the circulation of AIV in turkey transaction chains in LBMs. Therefore, our study aims to investigate turkey trading practices and temporal and seasonal patterns of H5 and H9 infection and the risk factors associated with these infections in turkey at LBMs in Bangladesh.

## 2. Methodology

### 2.1. Ethical approval

The research was approved by both the Animal Experimentation Ethics Committee and the Ethics Committee at the Chattogram Veterinary and Animal Sciences University (Protocol: CVASU/Dir (R&E) AEEC/2015/751 and CVASU/Dir (R&E) EC/2015/1011, respectively).

### 2.2. Study location and surveillance strategy

We conducted longitudinal surveillance in LBMs in Dhaka city, with the highest population density (30,551 residents/km^2^) and LBM density (659 numbers) in Bangladesh (42). Turkey is a relatively new poultry species in Bangladesh, and these birds were not usually found in all LBMs in Dhaka. Based on the regular selling of turkey birds, we selected two LBMs, Railway Market, Tejgaon, and Kaptan Bazar in Dhaka, for this study. We considered vendors who sell turkey at Railway Market, Tejgaon (LBM-1) is a mixed (Wholesale and retail) business type, and vendors at Kaptan Bazar (LBM-2) is a retail business type in our study based on the turkey bird selling practices. We weekly visited these two LBMs between May 2018 and Sept 2019. Every week, we sampled an average of 7 (min = 3, max = 14) birds in the LBM, depending on the availability of turkeys that week in that LBM, and the resulting sample size varied due to the availability of the birds and the traders’ preferences.

### 2.3. Biological samples and data collection

We collected cloacal and oropharyngeal swab specimens from each selected turkey using sterile cotton swabs and pooled them in a 1.8 mL sterile cryotube containing 1 mL of viral transport media (VTM) as previously described (43). We collected a total of 846 swab samples from 423 birds, including both juvenile and adult birds, over the course of 17 months at two LBMs. The team wore PPE (personal protective equipment), which included an N95 respirator, gloves, and goggles, and restrained the birds physically before taking samples following WHO guidelines (44). On the day of sampling, we transported specimens to the laboratory, and the samples were kept at −80°C freezer until laboratory analysis. We assessed the health condition of the birds and recorded the symptoms and demographic data (45, 46). We sampled dead birds if we found them at vendors and informed the vendors if they had any dead birds to keep for our sampling. We recorded the necessary information for individual vendors, bird health status, source of turkey, and selling practices related to hygiene and sanitation using a structured questionnaire.

### 2.4. Qualitative approach

In the qualitative investigation, we collected data using multiple approaches, including observation research, transect walks, and informal interviews, as described previously (47, 48). The trained field team observed the demand, price, trading patterns, and handling of sick birds. The transect walks approach was used to identify the vendors and middlemen selling and trading turkeys. The field team led multiple transect walks throughout the LBMs and stopped for informal interviews with vendors and middlemen. For 17 months in each LBM, the team conducted monthly informal interviews with vendors and middlemen, where they performed observations and face-to-face interviews in the early mornings at informants’ locations, generally near their shops. Informal interviews with turkey vendors/intermediaries were done until the team reached data saturation when no new information surfaced from various participants (49). The team established a rapport with the informants to increase the quality of the information provided. Throughout the research, the team used ethnographic diaries to capture daily comprehensive field notes, which aided in contextualizing the findings.

### 2.5. Laboratory analysis

We tested the pooled oropharyngeal and cloacal swab samples from each bird separately for the presence of the AIV viral Matrix (M) gene. We utilized the magnetic bead-based RNA isolation method to extract RNA using the MagMAXTM-96 AI/ND Viral RNA Isolation Kit (Applied BiosystemsTM, San Francisco, CA) in a KingFisherTM Flex 96-well robot (Thermo ScientificTM, Waltham, MA) following the manufacturer’s instructions (19). As a first step, we examined the swab samples for the presence of the M gene using real-time reverse transcription PCR (rRT-PCR) with reference primers and probes, as reported by (50). The samples that tested positive for the M gene were then subtyped for the H5, H7, and H9 using hemagglutinin gene-specific primers and probes in the rRT-PCR assay (51). Among M gene-positive samples, we classified them as AIV HA/untyped those negative for H5, H9, and H7.

### 2.6. Statistical analysis

We retrieved the data on the number of turkey birds per district from the agricultural census 2019, Bangladesh Bureau of Statistics (26). We collected the shape file from freely available DIVA-GIS^1^ (52). We produced the choropleth map for turkey population density. We tracked the route of turkey movements from source district farms to the point of selling LBM in ArcGIS 10.4 software using standard procedures as described (53). The number of trades from source districts to Dhaka between May 2018 and September 2019 was visualized using a Sankey diagram. We utilized the line graphs to determine whether there is any relationship between the number of trades from the source to Dhaka, the distance of the source district from Dhaka, and the turkey density in that district. In both the winter and summer seasons, we calculated the median distance traders travel to transport turkeys to Dhaka. We used the Chi-square test to see if there was any variation in the median distances between the two seasons. We determined the pattern of viral circulation from May 2018 to September 2019 by calculating the weekly and monthly proportion of AIV and subtypes. We used exploratory analysis to determine the pattern of viral circulation across seasons, health conditions, market types, and years. The value of Cramer’s V was then computed to assess if the season, health status, business type, and years may add multicollinearity to the multivariable model. In the multivariable model, we included the variables with Cramer’s V values less than 0.50 (19). For the multivariable analysis, “season,” “year,” “business type,” and “health status” were used as independent variables, and AIV, H5, and H9 (positive or negative) in each bird were considered as the dependent variable. Using generalized estimating equations (GEE), we fitted a logistic regression model to determine the risk factors associated with the “presence of AIV,” “presence of H5,” and “presence of H9” in turkey. For the analysis of longitudinal panel data, GEE is an effective approach, particularly when there is a possibility of correlation between the observations within each subject. The correlation matrix used in GEE models to describe the within-subject correlation is considered while estimating regression coefficients (54). We conducted all the analysis using RStudio version 4.1.2 (55). We used “lme4,” “geepack” and “tidyverse” packages for the analysis in R software.

## 3. Results

### 3.1. Turkey trading patterns

**Figure 1:**
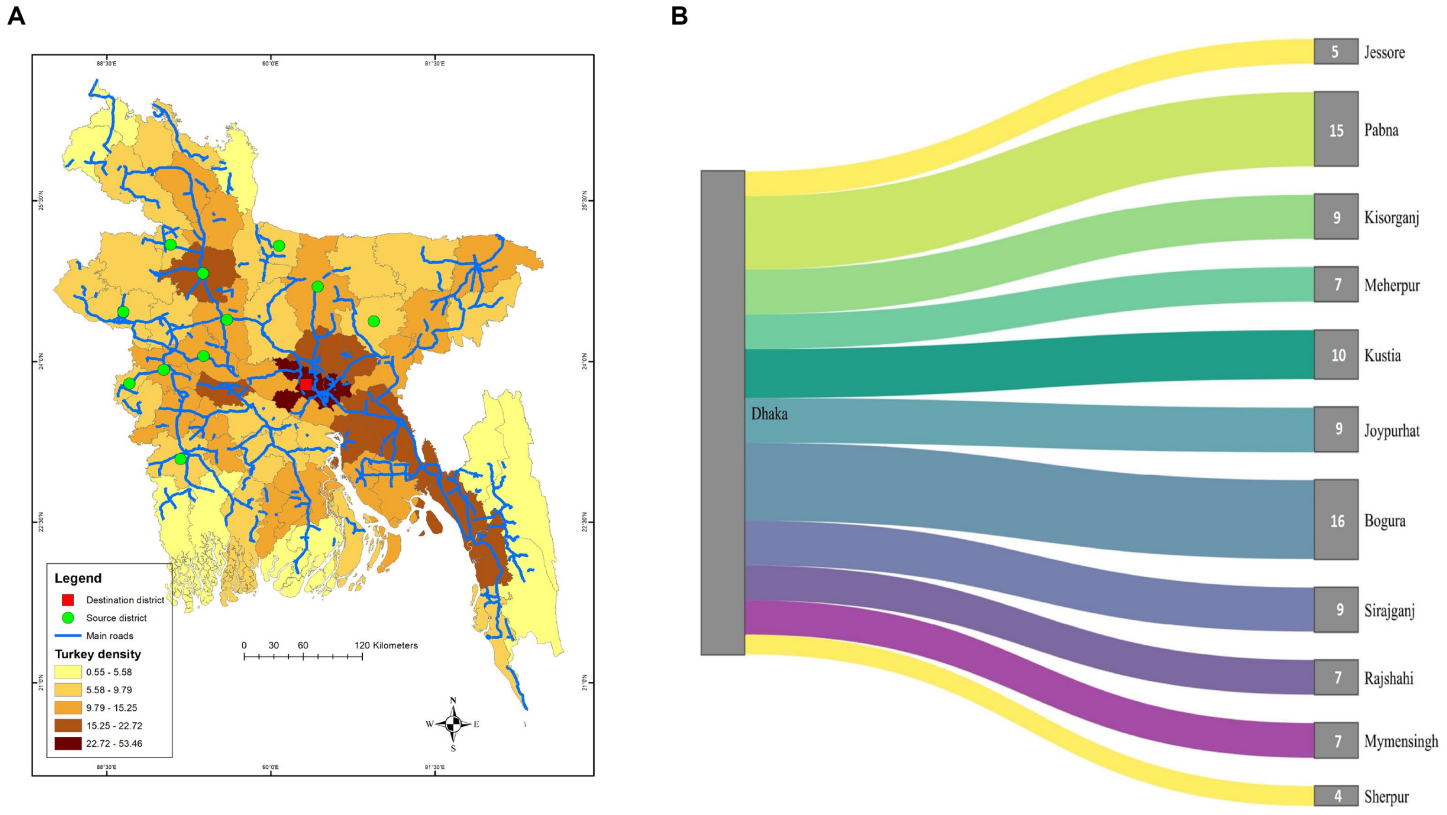
**(A)** Choropleth map of turkey density across the districts of Bangladesh plotted along with the trade routes of turkey from source districts (Green circle) to the LBMs (Red square) selected for the study. **(B)** Shankey diagram illustrating the number of trades from source districts to Dhaka in chosen LBMs for the study, where the thickness of links varies according to the number of trades conducted between the source and destination.

**Figure 2:**
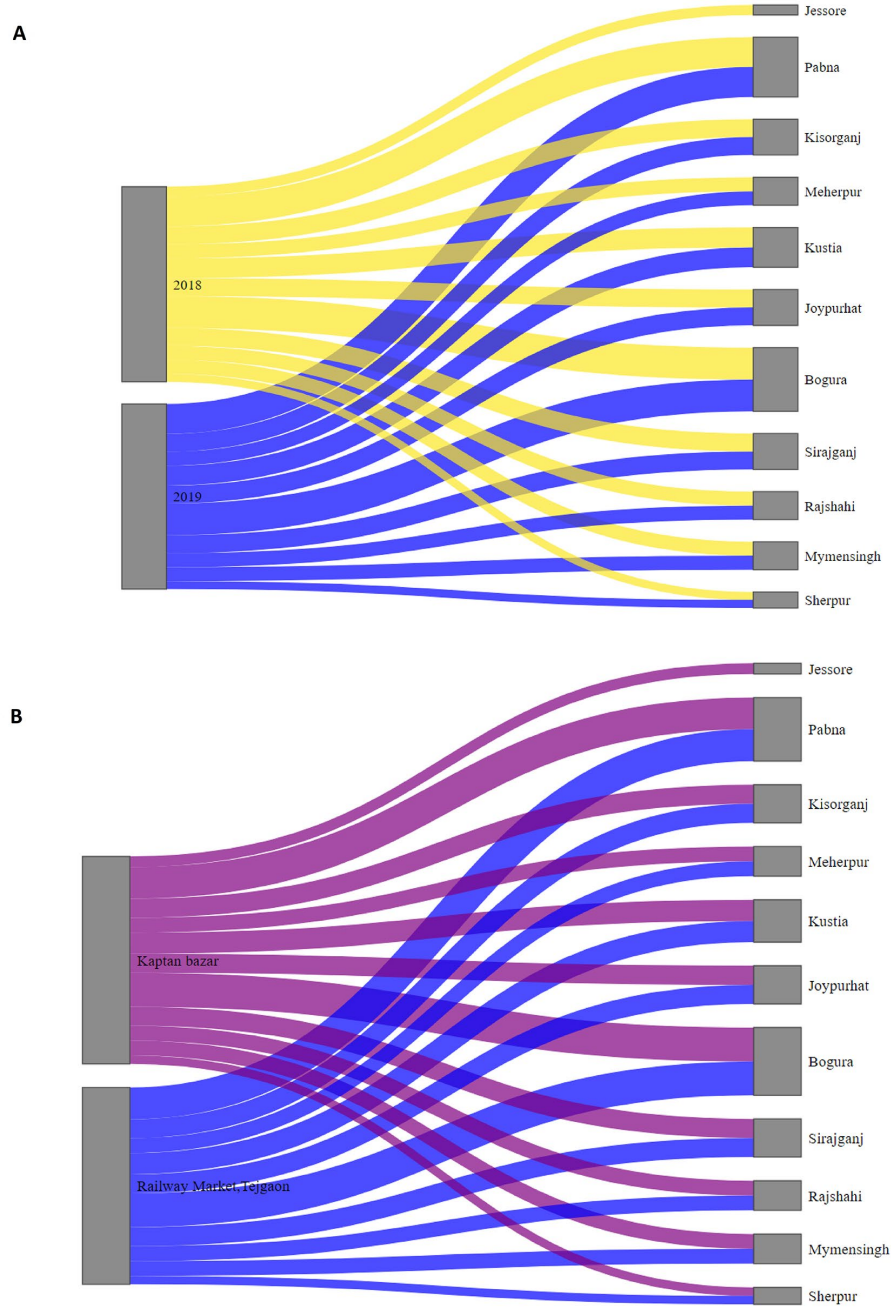
**(A)** Shankey diagram illustrating trade links from source districts to Dhaka in chosen LBMs for the study for 2018 (Yellow links) and 2019 (Blue links), where the thickness of links varies according to the number of trades conducted between the source and destination. **(B)** Shankey diagram illustrating trade links from source districts to Kaptan bazar (Purple links) and Railway Market, Tejgaon (Blue links), where the thickness of links varies according to the number of trades conducted between the source and destination.

**Figure 3:**
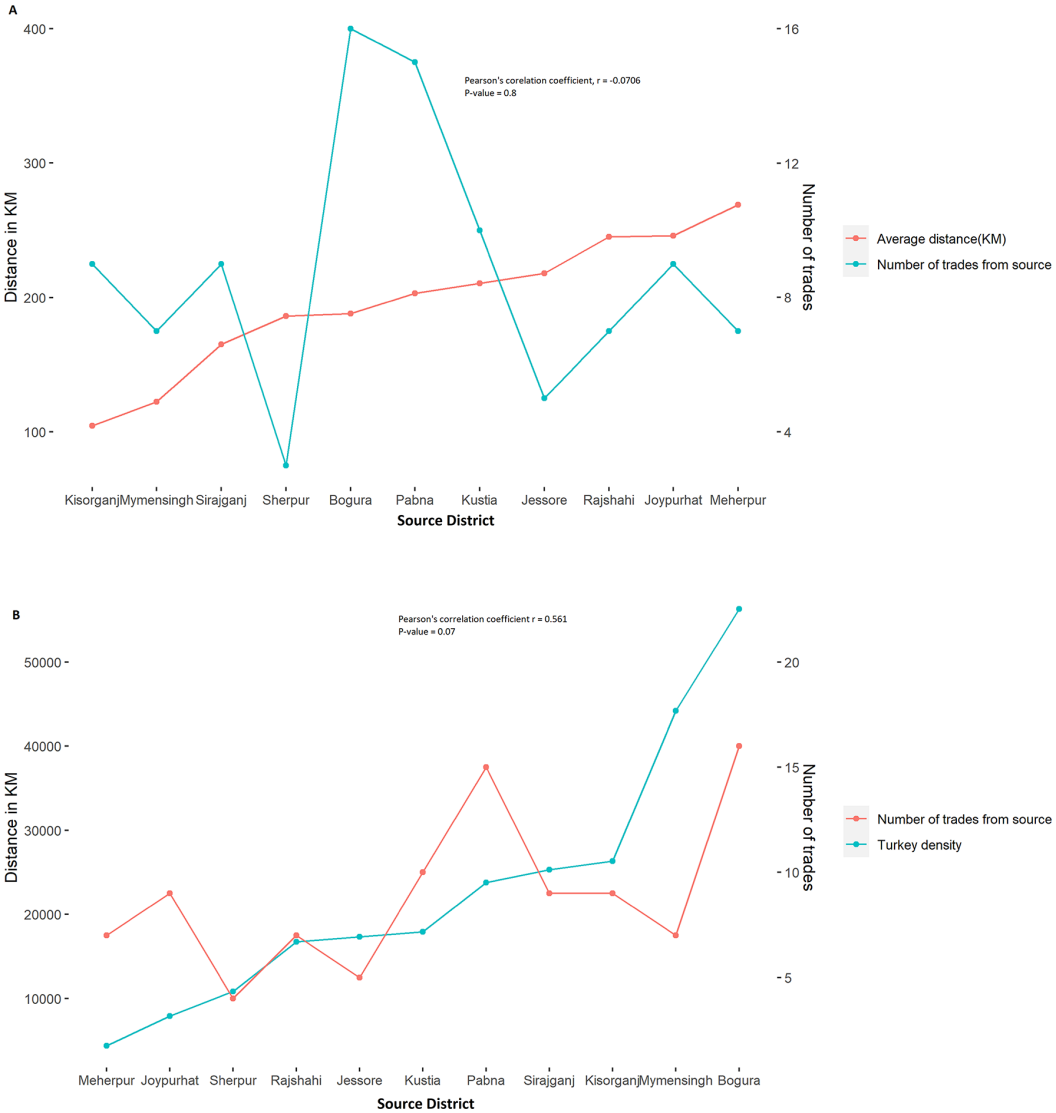
**(A)** Trend of the trade from the source to Dhaka and the trade pattern across the source’s distance. **(B)** The trading trend from the source to Dhaka and pattern of trade across turkey birds density of the source.

Figure 1A shows that the average distance of the source district from Dhaka and the number of trades does not follow any specific pattern. The Sankey diagram reveals that the most frequently traded turkeys in Dhaka LBM-1 and LBM-2 were from Bogura (16 times), Pabna (15 times), and Kushtia (10 times) during our study period (Figure 1B). There was no temporal distinction noticed in the source district of turkey, as most source districts substantially overlapped within the two years (Figure 2A). On the other hand, most source districts substantially overlapped, within the two LBMs, suggesting that both markets sold turkeys sourced from the same geographical areas (Figure 2B). The correlation (*r* = −0.0706) between the distance and the number of trades is also insignificant, with a value of *p* = 0.8 Figure 3A. On the other hand, From Figure 3B we can see that number of trades from the source district to Dhaka depends on the turkey density of the source district (*r* = 0.561, *p*-value = 0.07 < 0.1). We can see that Bogura has the highest density of turkey and the most frequent number of trades to Dhaka.

The majority (87.5%) of turkey movement was >122.3 km from the source and directed into Dhaka. The median distance that turkey was traded source district to Dhaka was 188 km (Q1 = 165, Q3 = 210, IQR = 45.5). Seasonal variation was observed in the median and average distance traveled by the turkeys during the winter and summer to be traded from the source district to Dhaka. Both the average distance (190.7 km) and median distance (203 km) in the winter season were significantly higher (*p* < 0.001) in the winter season than in the summer season (Supplementary Figure S1).

### 3.2. Observation study findings

#### 3.2.1. Turkey farming gains popularity

Our field team observed that during 2018, the demand for turkey was high in the market against the supply. Prices for turkey meat were high at the time, and the business was quite profitable. In 2018, predominantly affluent individuals and restaurants bought turkeys at a high price. As the general public became more interested in tasting different types of meat, the demand for turkey meat was high.

“Turkey meat sells for Tk.500–550 per kilogram. Since turkeys are large birds, selling one turkey produces more profit than selling smaller birds” (Vendor from LBM-2 in 2018).

Our field team observed that turkey supply also rose substantially at the markets end of 2018 and the start of 2019. According to middlemen at the LBM-1, as demand and profit were high, many poultry farmers, entrepreneurs, and unemployed youth began turkey farming. According to the discussion with the middlemen, turkey gained popularity among farmers since it could consume the same food as chicken along with grass and be kept in an open place. A turkey also matured quicker, allowing for more significant revenues in a shorter period of time.

“Turkey farming is gaining popularity among farmers because the bird can be raised easily in a free range system in backyard and scavenge on green grass and vegetables. As a result, farmers no longer rely solely on ready made feed from the markets” (Middleman from LBM-1 in 2019).

#### 3.2.2. A drop in demand

As a result of the increase in farming, the quantity of turkey in LBMs surged surprisingly. According to our observations and informal discussions with vendors and middlemen, we learned that although the supply of turkey was expanding considerably, demand halted, and the price began to decline. In Bangladesh, the appetite for turkey meat has not evolved to the extent it has for chicken and other types of fowl.

“The average weight of a turkey is 8–10 kg. Due to the high price of one bird based on its weight, low-income people are unable to purchase a whole turkey but can afford chicken. As a result of decreased demand, the price of turkey has dropped” (Vendor from LBM-1 in 2019).

In 2019 one of the vendors said,

“In LBMs, turkey birds were in high demand over the past few years, but that demand has recently decreased drastically. Nowadays, some people raise turkeys as a hobby and not for commercial purposes”.

We inquired as to why the demand for turkey meat was not increasing, and vendors and middlemen informed us that the general public was unaccustomed to the flavor of this new bird meat.

“Initially, consumers purchased turkey meat out of curiosity because they were not familiar with its taste compared to chicken and other types of poultry” (Vendor from LBM-2 in 2019).

According to our conversations with middlemen, turkey birds were diagnosed with infectious diseases, and mortality and morbidity were increasing, so in the middle of 2019, the farmers saw an economic collapse as the price of the birds also decreased, so they did not reshape the farms, and supply of the birds in LBMs dropped in the later part of 2019.

#### 3.2.3. Turkey trading and handling practices

We questioned the middlemen on the source of the turkeys, and most respondents said it was from the country’s northern and northwestern regions. Middlemen said that turkey density and other poultry trading routes were the primary cause for this section. Our team observed early morning to document how the turkey birds are exchanged. They found that turkey birds from various farms and other poultry species are transported on the same vehicle from the source to the LBM in Dhaka, and turkeys are not separately transported. Our team also noticed that sick turkeys are transported in the same vehicle as other turkeys and poultry.

One transporter said,

“We have leftover room in the vehicle after loading the turkey, so we mix turkeys from various farms and different species together for transportation”.

Our team observed the shops and pickups of the turkey birds to see how the vendors and middlemen handle the sick/dead birds. We watched that during the unloading of the turkey, if the middlemen noticed any sick or dead turkeys, they immediately slaughtered the turkey and sold it to restaurants.

“since, we paid for each turkey and that they are costly, we slaughter and sell sick turkeys at a discount rate to restaurants and the needy in order to minimize losses” (One Middleman from LBM-1 in 2019).

Our team also noticed that some sick turkeys died in the vendors’ shops, and some vendors threw them away in the dumpster of the LBMs, where other dead birds were also thrown away.

### 3.3. Pattern of the weekly and monthly proportion of AIV subtypes during 2018–2019 in Turkeys

**Figure 4:**
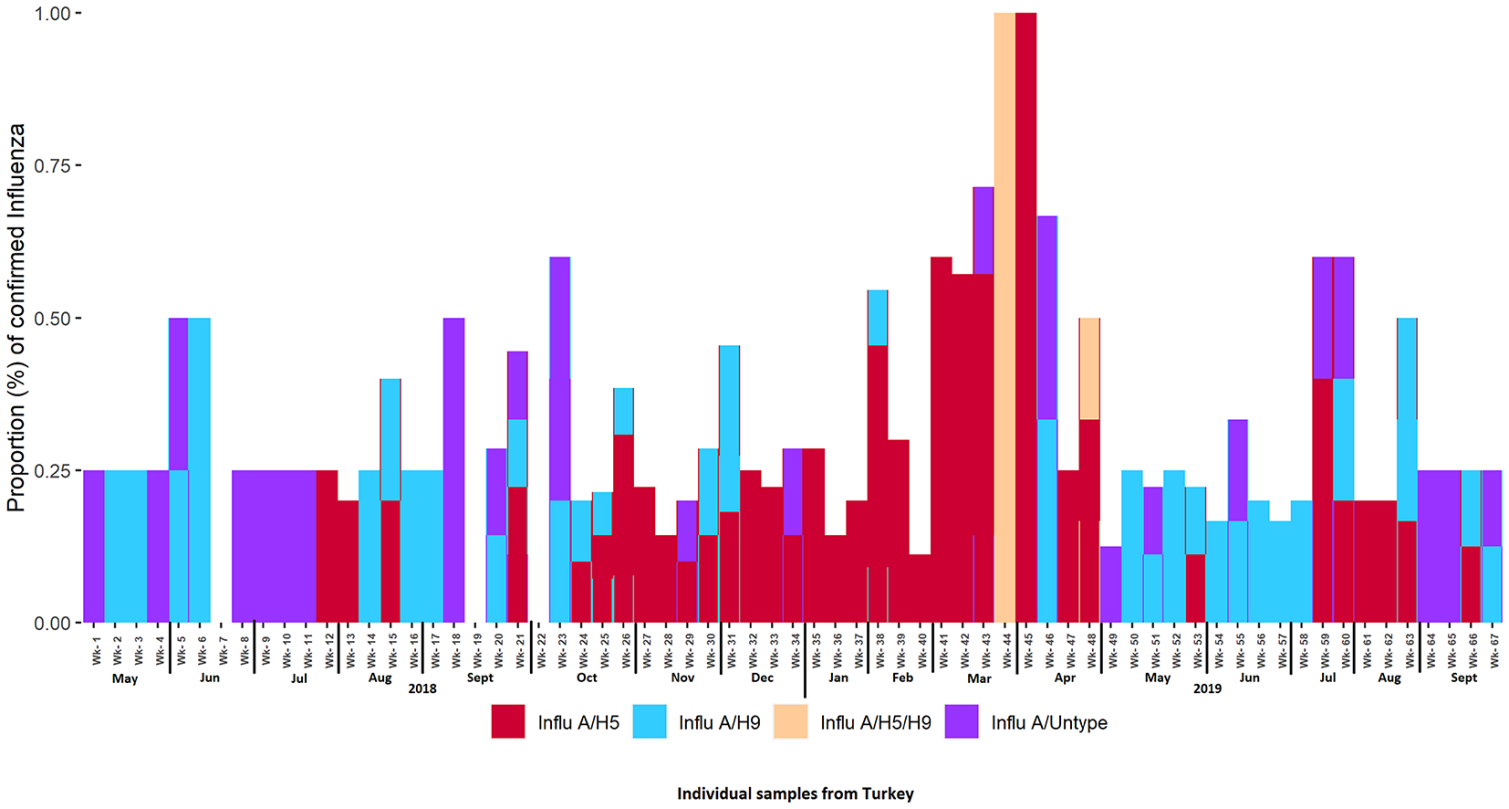
Proportion of confirmed AIV subtypes each week from May 2018 to September 2019.

**Figure 5:**
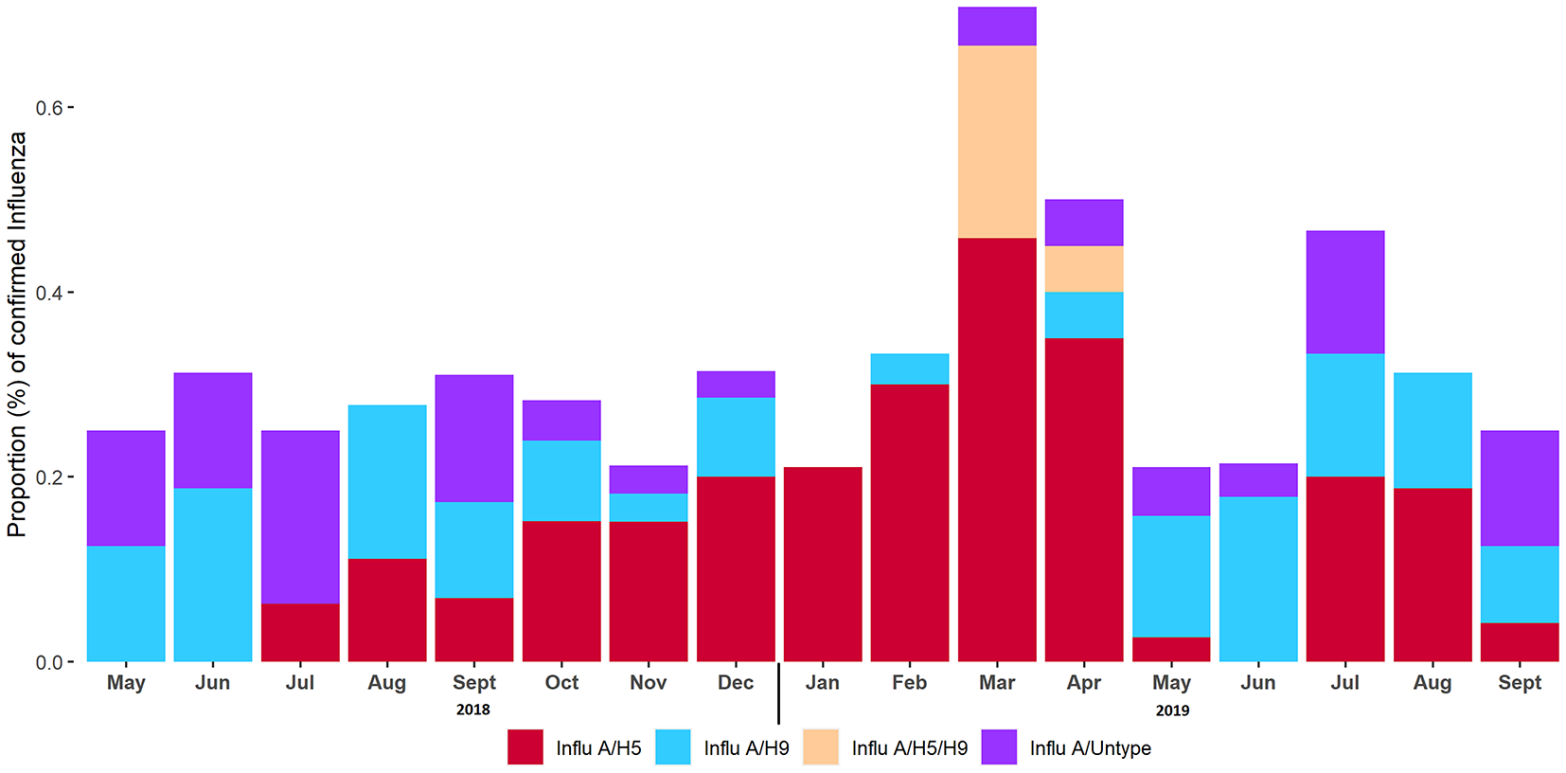
Proportion of confirmed AIV subtypes each month from May 2018 to September 2019.

The overall prevalence of AIV, H5, and H9 in turkeys was 31% (95% CI: 26.6–35.4), 16.3% (95% CI: 12.8–19.8), and 10.2% (95% CI: 7.3–13.1) respectively. We did not detect A/H7 in surveillance samples, and we also found co-circulation of H5 and H9 in turkeys. The overall prevalence for H5 and H9 co-circulation was 5.9% (95% CI: 3.9–8.7). The overall prevalence of H5 was higher than H9 in turkeys. Figures 4, 5 depict the weekly and monthly patterns of AIV subtype circulation in turkeys from May 2018 to September 2019. During the study period, AIV was detected each month in the LBMs, and March 2019 marked the peak of AIV circulation. On the other hand, in March (week 44) and April (week 48) of 2019, co-circulation of H5 and H9 was detected in turkeys.

H5 has been detected monthly throughout the research period, except for May–June 2018, and June 2019. We can also see that from week 24 through week 45, A/H5 was consistently detected every week. All the samples were detected as A/H5 during week 45 (April 2019) (Figure 4). We also observed that in LBM-2, H5 was detected consistently from Oct 2018 to Mar 2019 (Supplementary Figure S2) and from July 2018 to May 2019 in LBM-1 (Supplementary Figure S3).

In contrast, H9 was detected each month during the duration of the investigation, apart from July 2018 and January 2019. The highest proportion (50%) of H9 was in week 6 (June 2018). No virus was detected in weeks 7 (June 2018), 19 (September 2018), and 22 (October 2018). HA/untyped was also detected sporadically throughout the study period.

### 3.4. Seasonal transmission patterns of AIV and subtypes in turkey birds

#### 3.4.1. Prevalence of AIV, H5, and H9 in Turkey across the annual cycle in LBM

**Figure 6:**
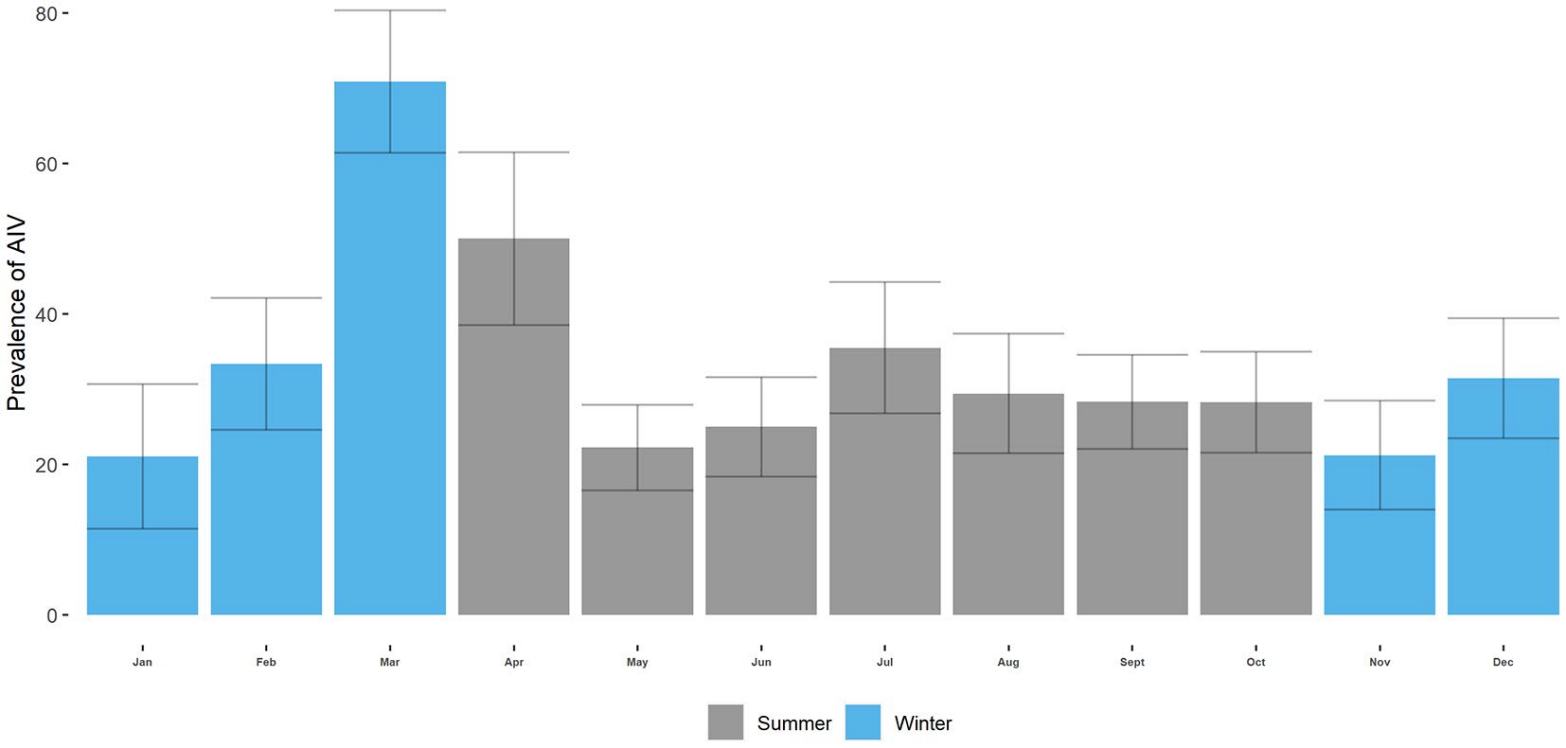
AIV prevalence in Turkey across the annual cycle at LBM.

We can see that the monthly prevalence of AIV ranged from 21 to 71%. The highest prevalence of AIV was in March (71%; 95% CI: 52–89), and the lowest was in January (21%; 95% CI: 2–40) (Figure 6). We can see that the circulation of AIV over the annual cycle shows no specific pattern or seasonality (value of *p* = 0.3) (Figure 6 and Supplementary Figure S4).

**Figure 7:**
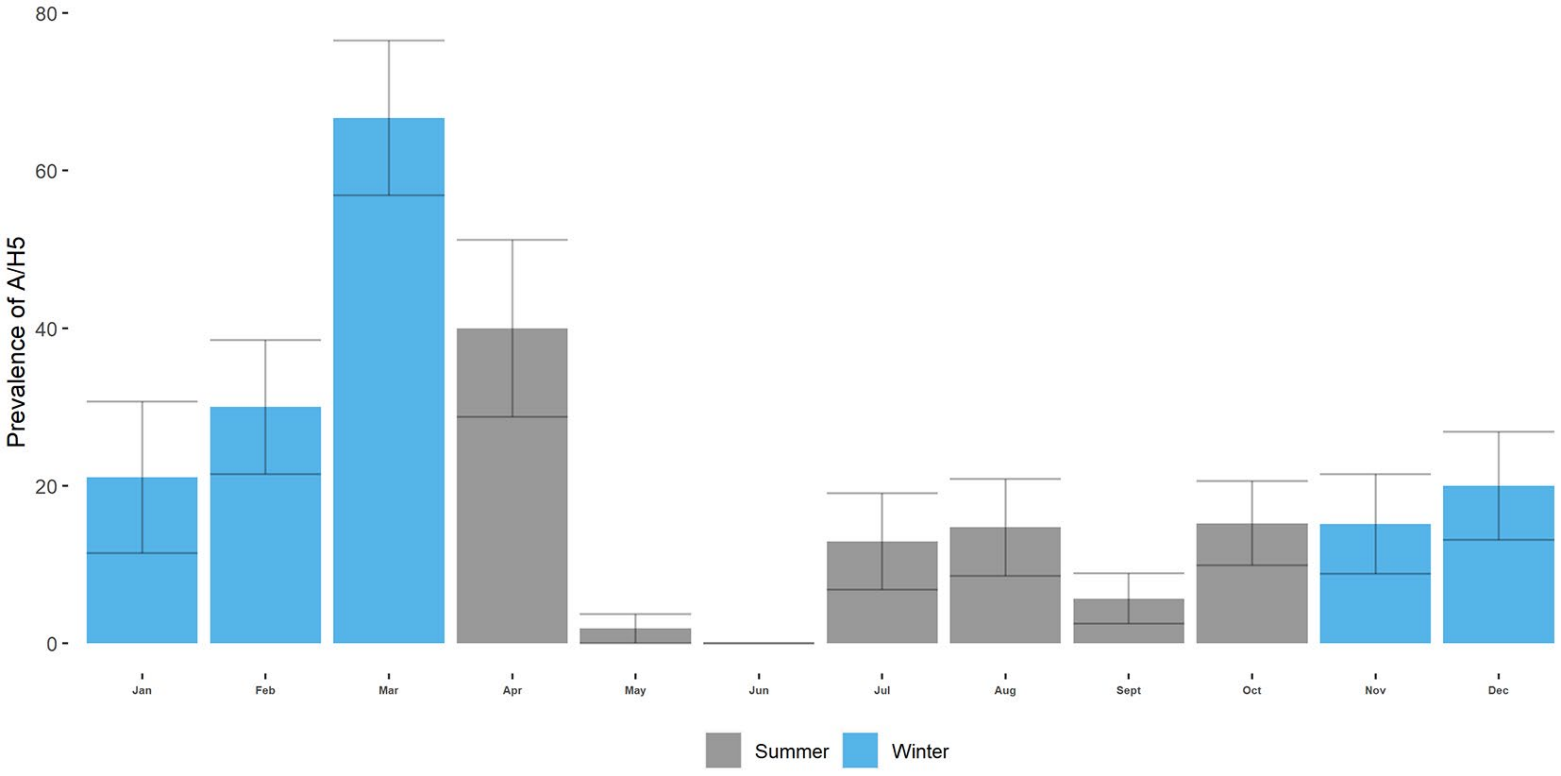
A/H5 prevalence in Turkey across the annual cycle at LBM.

**Figure 8:**
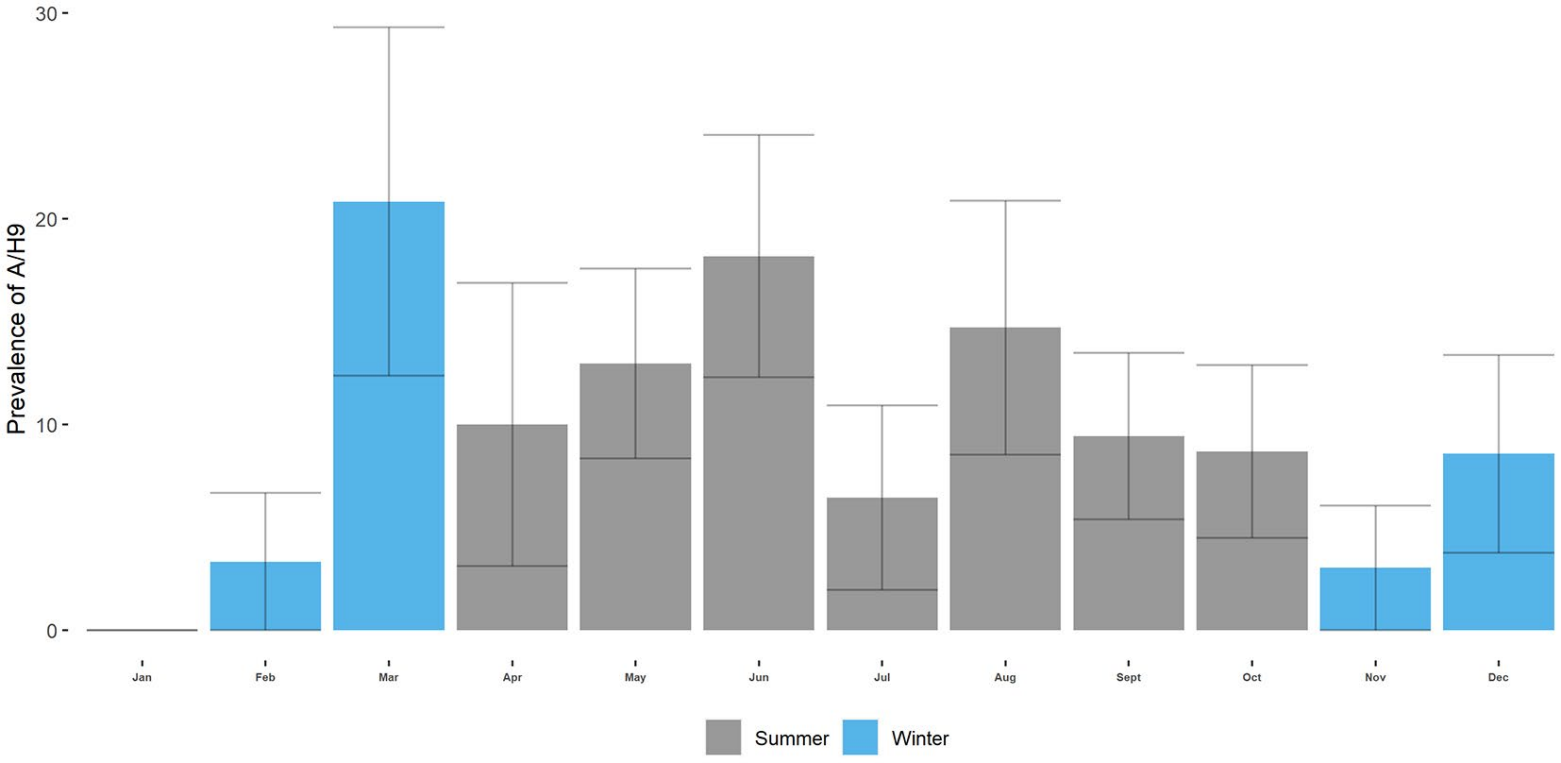
A/H9 prevalence in Turkey across the annual cycle at LBM.

The monthly prevalence of H5 ranged from 0 to 66.67%. The highest prevalence of H5 was in March (66.67%; 95% CI: 48–87), and the lowest was in June (Figure 7). We can see specific patterns in the circulation of H5 over the annual cycle. The prevalence of H5 in the summer (April–October) is lower than in the winter months (November–March) (*p* < 0.001; Figure 7; Supplementary Figure S5). The monthly prevalence of H9 ranged from 0 to 20.83% (Figure 8). The overall prevalence of H5 was higher than H9 in turkeys. The highest prevalence of H9 was in March (20.83%; 95% CI: 4.2–37.43), and the lowest was in January. However, in the circulation of H9, no specific pattern is seen across the annual cycle (Figure 8; Supplementary Figure S6).

### 3.5. Prevalence of AIV, H5 and H9 by health status

**Figure 9:**
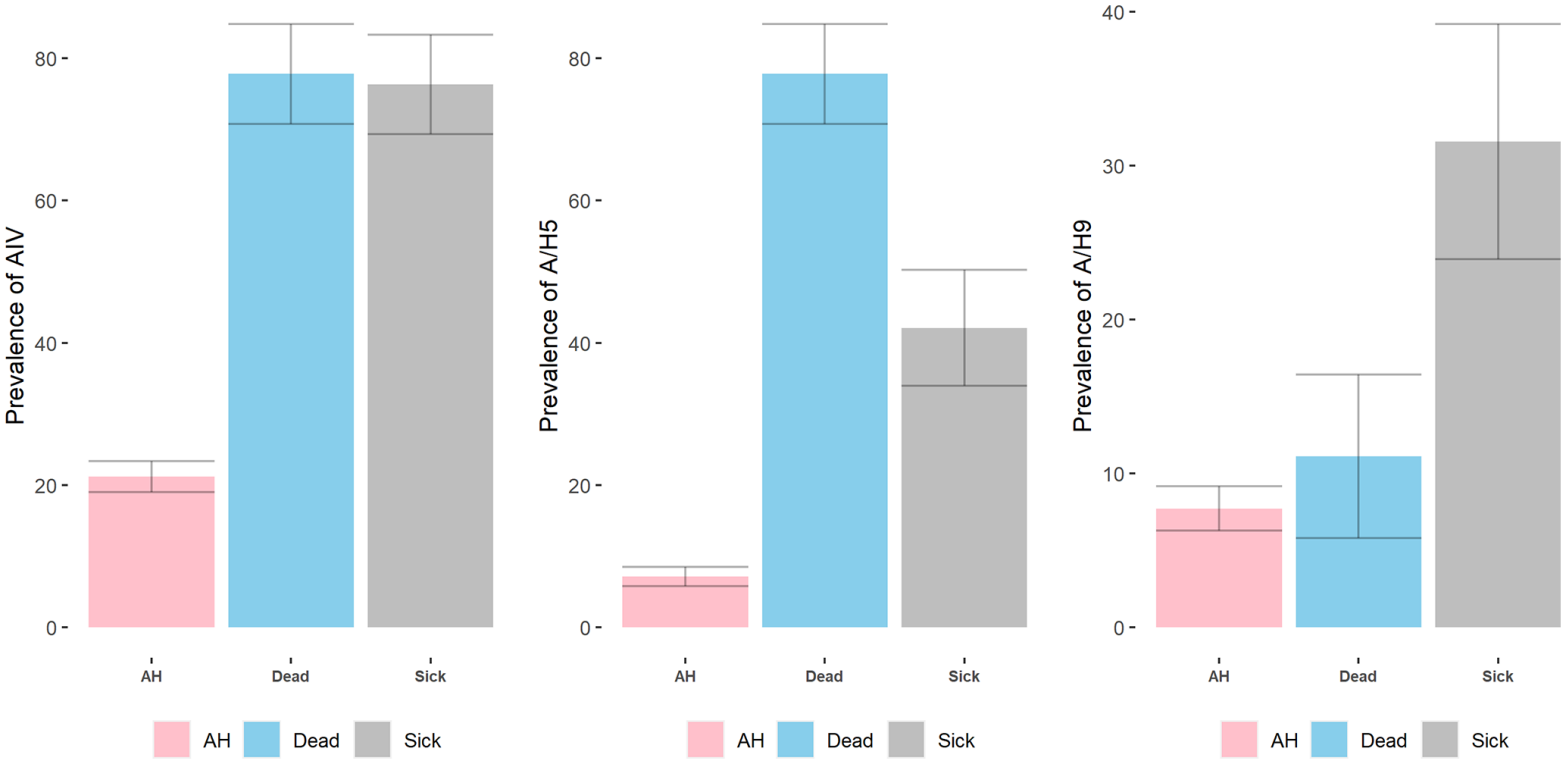
Prevalence and 95% CI of AIV, A/H5, and A/H9 across health status.

The prevalence of AIV in apparently healthy birds is 21.20% (95% CI: 16.9–25.4), which was much higher in sick (76.3%; 95% CI: 63–90) and dead birds (77.78%; 95% CI: 64–91.6) (Figure 9). A similar pattern was also observed in the circulation of H5. In apparently healthy turkeys, only 7.2% of birds were infected. However, in sick turkeys’ the prevalence of H5 was 42.11% (95% CI: 26–57.9). H5 was detected highest in the dead turkeys, where 77.78% (95% CI: 64–91.6) of the samples from dead turkeys were positive for H5. On the other hand, H9 was primarily detected in sick birds (31.6%; 95% CI: 16.5–46.5) than apparently healthy (7.73%; 95% CI: 4.9–20.5) or dead birds (11.1%; 95% CI: 0.68–21.5).

### 3.6. Prevalence of AIV, H5, and H9 by the business status of the vendor

**Figure 10:**
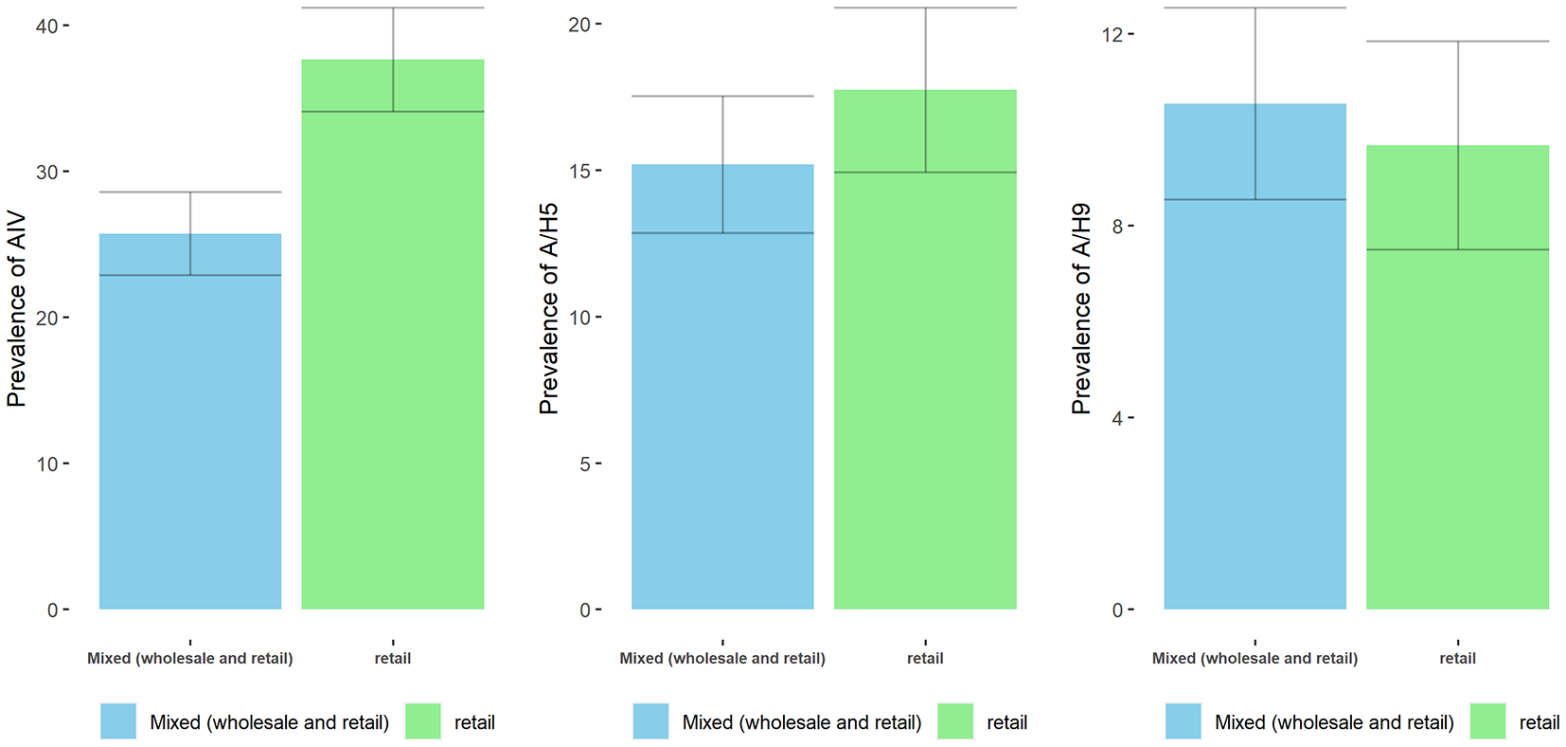
Prevalence and 95% CI of AIV, A/H5, and A/H9 across the business type of vendor.

Prevalence of AIV was higher among the samples collected from vendors retail (37.63%; 95% CI: 30.6–44.6) business type than those with mixed (wholesale and retail) businesses (25.7%; 95% CI: 20.1–31.3) (Figure 10). Similarly, the prevalence of H5 was higher among the samples collected from vendors retail (17.7%; 95% CI: 12.2–23.2) business type than those with mixed (wholesale and retail) businesses (15.2%; 95% CI: 10.6–19.8). However, in H9, the opposite pattern was observed. Prevalence of H9 was higher in mixed (wholesale and retail) businesses (10.6%; 95% CI: 6.6–14.5) than in retail businesses (9.7%; 95% CI: 5.4–11.9).

### 3.7. Prevalence of AIV and H5, and H9 subtypes in Turkeys by year

**Figure 11:**
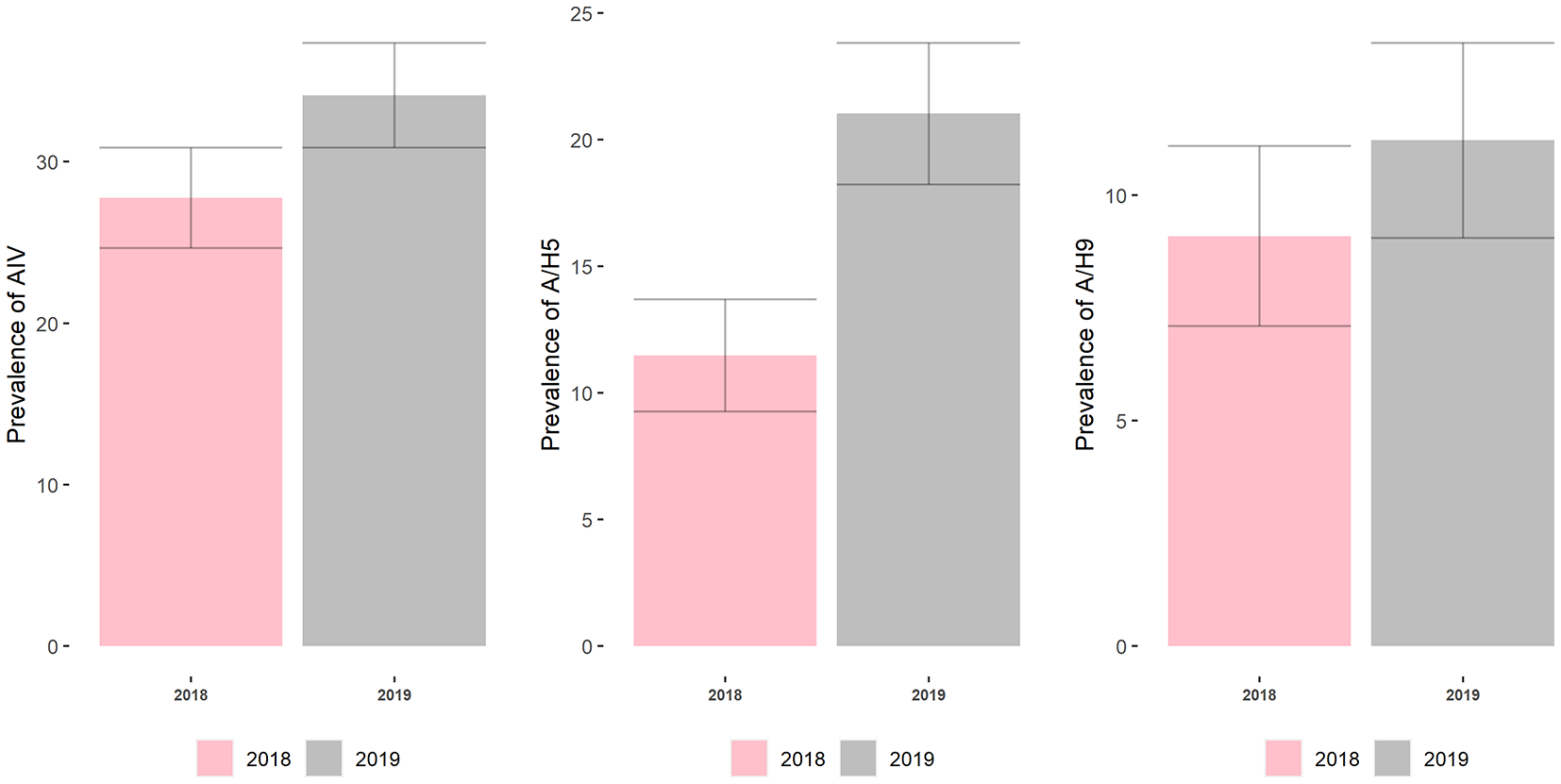
Prevalence and 95% CI of AIV, H5, and H9 subtypes across the year.

The prevalence of AIV, A/H5, and A/H9 was higher in 2019 than in 2018 (Figure 11). The prevalence of AIV in turkeys was 27.8% (95% CI: 21.7–33.9) in 2018, which increased to 34.1% (95% CI: 27.7–44.5) in 2019. Similarly, the prevalence of H5 and H9 in turkeys was 11.5% (95% CI: 7.2–15.8) and 9.1% (95% CI: 2.0–13.0), respectively, in 2018, where it increased to 21.0% (95% CI: 15.5–26.5) and 11.2% (95% CI: 76.0–15.4) respectively in 2019.

### 3.8. Risk factors of H5 and H9 circulation in LBM

According to our hypothesis, we took season, year, health status, and business type in a multivariable logistic regression model using the GEE approach. We calculated Cramer’s V (Supplementary Figure S7) to check multicollinearity between the independent variable. However, the association of variables, as evaluated by Cramer’s V, was less than 0.40, indicating no multicollinearity among them. The model for AIV shows that the vendor’s business type and the bird’s health status influence the circulation of AIV. The odds of AIV increased when the business type of the vendor was retail (OR: 1.71; 95% CI: 1.12–2.62) rather than mixed. On the other hand, the chance of detection of AIV was much higher in sick (OR: 10.77; 95% CI: 4.31–26.94) and dead birds (OR: 11.33; 95% CI: 4.30–29.89) than in healthy ones.

From the model for H5, we can see from Table 1 that season and health status are the associated risk factor with the presence or absence of H5 in turkeys. The odds of H5 infection were 5.59 (95% CI: 2.76–11.70) times higher in the winter than in the summer season. On the other hand, the detection of H5 was 65.29 (95% CI: 27.55–154.73) times higher in dead birds and 8.70 (95% CI: 3.89–19.46) times higher in sick birds than apparently healthy ones. On the other hand, we found that only health status is a significant factor in the model of H9. The detection of H9 in sick birds is likely 6.59 (95% CI: 3.20–13.57) times higher than in the apparently healthy birds.

**Table 1:** Results from logistic regression using GEE.

Predictors		AIV			H5			H9		
		Odds ratios	CI	*p*	Odds ratios	CI	*p*	Odds ratio	CI	*P*
Season	Summer	Ref	–	–	Ref	–	–	Ref	–	–
	Winter	1.27	0.79–2.02	0.321	5.59	2.67–11.70	**<0.001**	0.44	0.16–1.25	0.125
Year	2018	Ref	–	–	Ref	–	–	Ref	–	–
	2019	1.35	0.82–2.21	0.236	1.82	0.83–3.97	0.134	0.96	0.49–1.92	0.918
Health status	Apparently healthy	Ref	–	**–**	Ref	–	–	Ref	–	–
	Dead	11.33	4.30–29.89	**<0.001**	65.29	27.55–154.73	**<0.001**	1.73	0.28–10.57	0.554
	Sick	10.77	4.31–26.94	**<0.001**	8.70	3.89–19.46	**<0.001**	6.59	3.20–13.57	**<0.001**
Business type	Mixed	Ref	–	**–**	Ref	–	–	Ref	–	–
	Retail	1.71	1.12–2.62	**0.013**	1.12	0.57–2.19	0.743	0.68	0.35–1.34	0.263

The bold values in table 1 indicates *p*-value < 0.05.

## 4. Discussion

This study provides evidence for the persistence of AIV with H5 and H9 subtypes in turkey birds in LBMs over a 17-month surveillance period in Dhaka city in 2018–2019. However, None of the samples were positive for the H7 subtype. Previous studies showed that trading patterns differed depending on the sort of poultry traded (56). Exotic broilers are the most common meat type of chicken sold, and they come from both farms and others near the studied LBMs. Sonali [crossbred of Fayoumi female and RIR (Rhode Island Red) male], Deshi (the breeds of chickens native to Bangladesh) (57). On the other hand, domestic ducks were mainly acquired from other LBMs and purchased from a greater distance (58). Consequently, understanding the trading pattern of turkey is critical. Our study shows that turkey trade routes to northern Dhaka are unidirectional and encompass the northwestern and southern regions of Bangladesh. Despite the high turkey density in Chattogram, Cumilla, and Sylhet (Figure 10), turkey trade routes were unavailable from the country’s southeast and east to northern Dhaka. A similar situation occurred for Broiler, Sonali, and Deshi chicken trade routes and catchment areas (41). The catchment area of these chickens does not overlap with the cities of Dhaka and Chattogram (41). Similarly to our findings in turkey’s trading route, the majority of Deshi, Sonali, and Ducks are sourced from the country’s center and northwestern areas (41).

Our study findings revealed that the density of turkey in a source district has a significant impact on the quantity of trading that occurs between the source district and the LBM in northern Dhaka. We also determined that the source of turkey in our study overlaps substantially across LBM, and over time, comparable findings have been reported for other poultry species (41), indicating that poultry trade routes remain constant over time and across LBM not only for turkey but also for other poultries depending on poultry density in the source district (Figure 11).

We found that most of the turkeys movement to Dhaka was >122.3 km. The median distance that turkeys were traded from the source district to Dhaka was 188 km, similar to the trading distance of duck and Deshi chickens but way higher than broiler movements in Bangladesh (41). On the other hand, poultry transported in countries like Vietnam, Cambodia, and Thailand were only >10 km from source to destination, and the median distance traveled by poultry in these countries was only 70 km (59). The long-distance travel of turkey birds may increase the likelihood of AIV transmission during the trading movement in Bangladesh as the latent period associated with AIV infection in poultry is thought to be short, lasting less than a day (60). Moreover, from the observation study, we found that the turkeys are caged with other poultry and turkeys from various farms during trading. According to the informal discussion, farmers want to sell the birds as soon as possible if they notice they are sick or dying to reduce economic losses. In that case, AIV-infected birds may be traded in the LBM from the farm. Since we collected samples from the turkeys soon after the birds arrived at the LBM, the turkeys probably contracted AIV along the trade route rather than at the LBM. Previous studies also showed that viral shedding in markets was most likely caused, at least in part, by infection happening before poultry arrived at markets, during transit, and potentially during collection from farms (56).

The prevalence of AIV in turkeys in our study was higher than in other poultry species in the LBMs of Bangladesh. The prevalence of AIV in poultry species (chickens, ducks, quails, and pigeons) ranged from 12 to 28% in the LBMs (33). This could be because turkeys are highly susceptible to AIV infection (61, 62). When judging the health of turkeys, good hygiene, housing, brooding, and stocking density are all essential (63). Also, like broiler chickens, turkeys require high protein and other nutrients in their diets (64). So, a lack of proper knowledge of the turkey-rearing system can reduce turkey immunity and increase AIV transmission. The prevalence of H5 in turkeys was higher in our study than in H9. Turkeys are more susceptible to HPAI virus infection than other poultry species (8, 9). Furthermore, hence a greater prevalence of H5 was reported. An H5N1 outbreak was also reported in turkey flocks in Bangladesh in 2017 (15). Our study also detected H9 and A/untyped viruses in turkeys. LPAI viruses like H6N1 and H7N6 have been detected in turkeys in countries like France, Chile, and the Netherlands (62, 65, 66). The outbreak of the H7N3 virus was also detected in turkeys in the Netherlands (65). So, these subtypes may be circulating in turkeys within the LBMs of Dhaka, and further intensive surveillance is needed to detect these viruses.

We found no seasonality in the circulation of AIV in turkeys, with a peak in March 2019. On the other hand, Berry, Rahman (67) found AIV displayed weak seasonality, with moderate year-round transmission and a peak in April in LBMs in poultries. We also detected strong seasonality in the circulation of A/H5 and no seasonality in the circulation of A/H9. Seasonality in the circulation of AIV and subtypes may vary based on environmental samples or samples from other species. These findings pertain only to turkeys. We also found that season is a significant factor for the circulation of H5 but not for H9. The odds of H5 were higher in the winter than in the summer season. In Bangladesh, the temperature lowers significantly during the winter season. Moreover, several studies have shown that the likelihood of AIV outbreaks increases in lower temperatures (68–70). The reasons behind this could be attributed to the fact that lower temperatures may facilitate the replication and survival of the virus (71). On the other hand, several bacterial infections, such as *E. coli, Salmonella*, and infectious bronchitis virus (IBV), have been shown to be more prevalent in colder months in turkey birds (72, 73). The presence of other infectious agents or co-infections in the same bird could also increase the risk of AIV transmission. For instance, bacterial or parasitic infections, as well as other viral infections, could weaken the immune system of the bird and make them more susceptible to AIV infection (74).

We also found that health status is a significant factor for both H5 and H9 infection in birds. We showed that the sick turkeys have a higher likelihood of H5 and H9 detection. The HPAI H5N1 and the LPAI H9N2 have been linked to cases of illness in birds (75, 76). On the other hand, dead turkeys have a higher likelihood of H5. It is possible that the HPAI AIV was the cause of the birds’ deaths (77). A/H5 was detected in seemingly healthy turkeys. Previous research has suggested that H5 seropositivity without any HPAI clinical symptoms or mortalities may result from LPAI H5 strain infection in backyard chickens (78). In contrast, the existence of LPAI H5N2 was discovered across Bangladesh (79), along with the spread of other LPAI H5 viruses (H5N2, H5N3, and H5N8) in the Asia (80, 81). Alternatively, viral evolution might have lowered HPAI H5 pathogenicity, or the turkeys could have gained a reduced vulnerability to clinical illness due to cell-mediated immunity.

Turkey farming, which began as a promising effort, is undergoing a difficult time in Bangladesh, with the sector’s volume dropping rapidly. In less than 2 years, the country’s turkey farms shrank to a fraction of their former size (82, 83). From our informal discussion, we found that, along with the decrease in the price of turkey meats, infectious diseases were another reason for the lower supply of turkey in the market. AIV can cause significant morbidity and mortality in turkey populations, and outbreaks of AIV in turkeys can result in lower supplies of turkey due to culling and decreased production (13, 84). Our team noted that the foremost obstacles to the turkey industry are volatile market prices, poor feed at a higher cost, and a lack of adequate marketing facilities. Lack of public awareness and scarcity of advertising, lack of veterinary services, training, and skilled personnel are also severe issues (85). There is a considerable scope of turkey rearing in Bangladesh, as turkeys can be reared in a free-range farming system (4). It has good prospects and a new dimension in the poultry sector (86). The suitability of climatic conditions, and the availability of natural feed and manpower can make this sector profitable, especially for the poor and marginal farmers (64).

## 5. Conclusion

This study explored how turkeys movement patterns varied over time and season from farm to end selling points at the LBM. Long-distance bird movement may play a role in the transmission of AIV viruses in birds. We found continuing circulation of AIV subtype H5 and H9 in turkeys in LBMs in Dhaka during 17 months of surveillance in 2018–2019. We observed the selling of sick turkeys carrying H5 and H9, which underscores the risk of transmitting the virus to other species of bird selling at LBMs and spillover of humans. Taking the appropriate corrective measures, turkey farming might evolve into a profitable commercial business that plays a significant role in the poultry sector by supplying protein, generating income, and creating employment opportunities, thereby improving the livelihoods of rural people.

## Data availability statement

The original contributions presented in the study are included in the article/supplementary material, further inquiries can be directed to the corresponding author.

## Ethics statement

The studies involving human participants were reviewed and approved by Ethics Committee at the Chattogram Veterinary and Animal Sciences University (Protocol: CVASU/Dir (R&E) AEEC/2015/751 and CVASU/Dir (R&E) EC/2015/1011, respectively). The participants provided their written informed consent to participate in this study. The animal study was reviewed and approved by Animal Experimentation Ethics Committee and the Ethics Committee at the Chattogram Veterinary and Animal Sciences University (Protocol: CVASU/Dir (R&E) AEEC/2015/751 and CVASU/Dir (R&E) EC/2015/1011, respectively). Written informed consent was obtained from the owners for the participation of their animals in this study.

## Author contributions

AI: conceptualization. AI, AM, and SI: field investigation. AI, AM, MEH, and SI: data curation. MS, MAS, MEH, and MR: laboratory analysis. AI and EA: formal analysis and wrote the initial draft. AI, MMH, TS, and MR: funding. MMH, TS, and MR: supervision. MEH, SI, MR, and MMH: review and edited the manuscript. All authors have read and approved the final version of the manuscript.

## Funding

The study was supported by the University Grant Commission (UGC) of Bangladesh through Chattogram Veterinary and Animal Sciences University (CVASU), grant number UGC/CVASU#06, and USAID Emerging Pandemic Threats PREDICT project (AID-OAA-A-14-00102) through EcoHealth Alliance.

## Conflict of interest

The authors declare that the research was conducted in the absence of any commercial or financial relationships that could be construed as a potential conflict of interest.

## Publisher’s note

All claims expressed in this article are solely those of the authors and do not necessarily represent those of their affiliated organizations, or those of the publisher, the editors and the reviewers. Any product that may be evaluated in this article, or claim that may be made by its manufacturer, is not guaranteed or endorsed by the publisher.

## Footnotes

^1^https://www.diva-gis.org/gdata
