# Supplementary material for: Annual trading patterns and risk factors of avian influenza A/H5 and A/H9 virus circulation in turkey birds (Meleagris gallopavo) at live bird markets in Dhaka city, Bangladesh
Source: Front Vet Sci. 2023 Jul 4;10:1148615. doi: 10.3389/fvets.2023.1148615 (PMC10352991; doi:10.3389/fvets.2023.1148615)

Figure S1: Seasonal Variation in Turkey median trading distance and average trading distance from source to Dhaka.

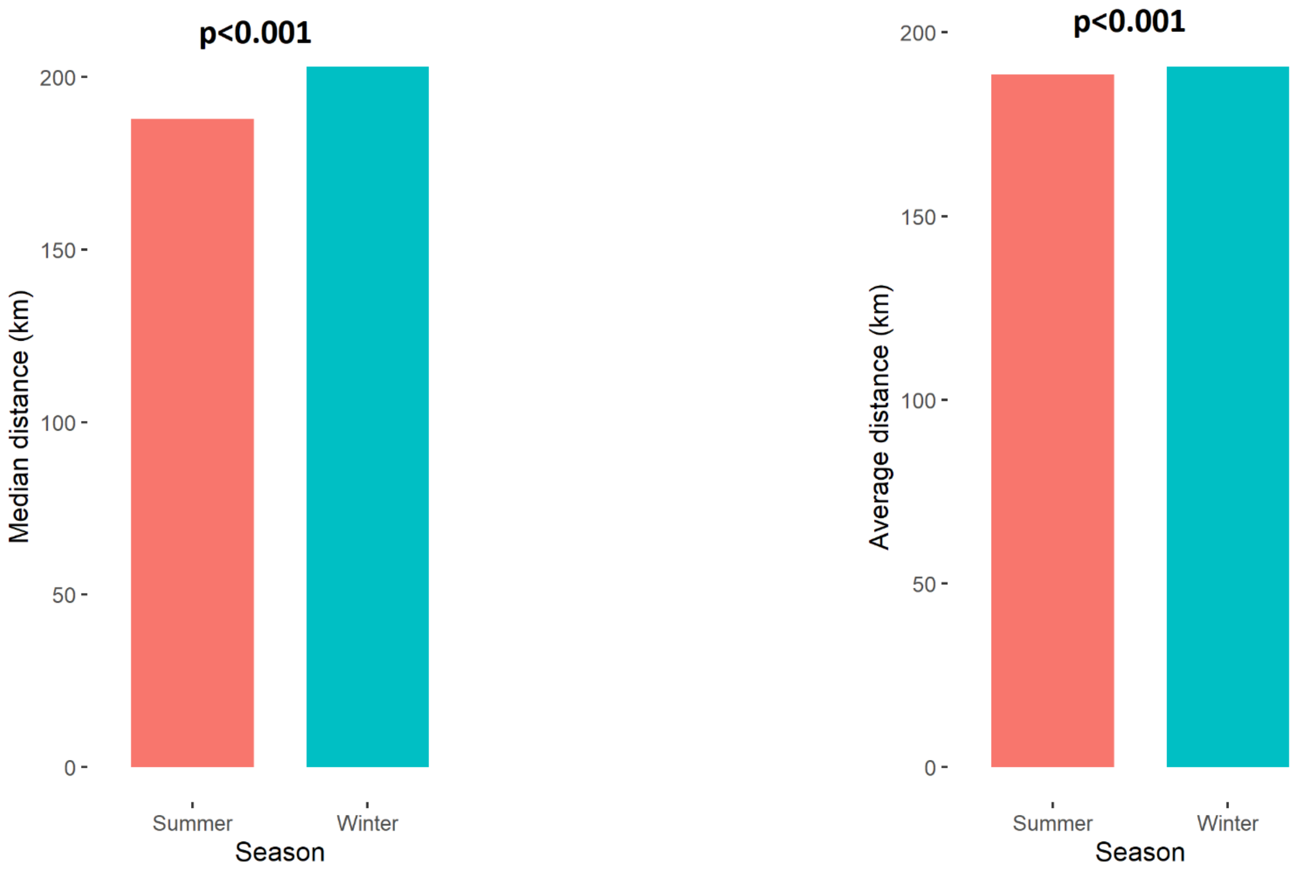

Figure S2: Proportion of confirmed AIV subtypes each month from May 2018 to September 2019 in Kaptan Bazar (LBM-2)

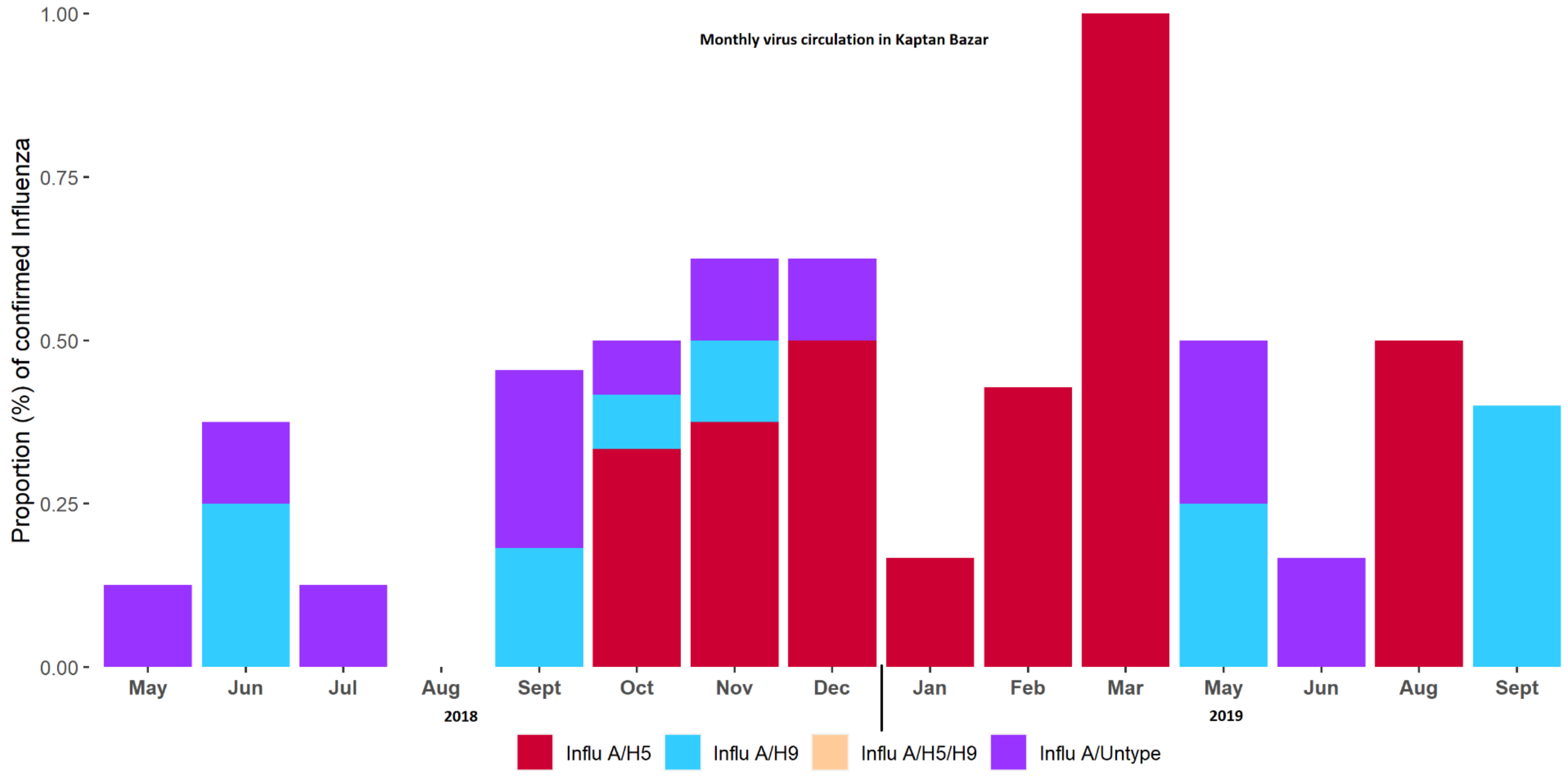

Figure S3: Proportion of confirmed AIV subtypes each month from May 2018 to September 2019 in Railway Market, Tejgaon (LBM-1)

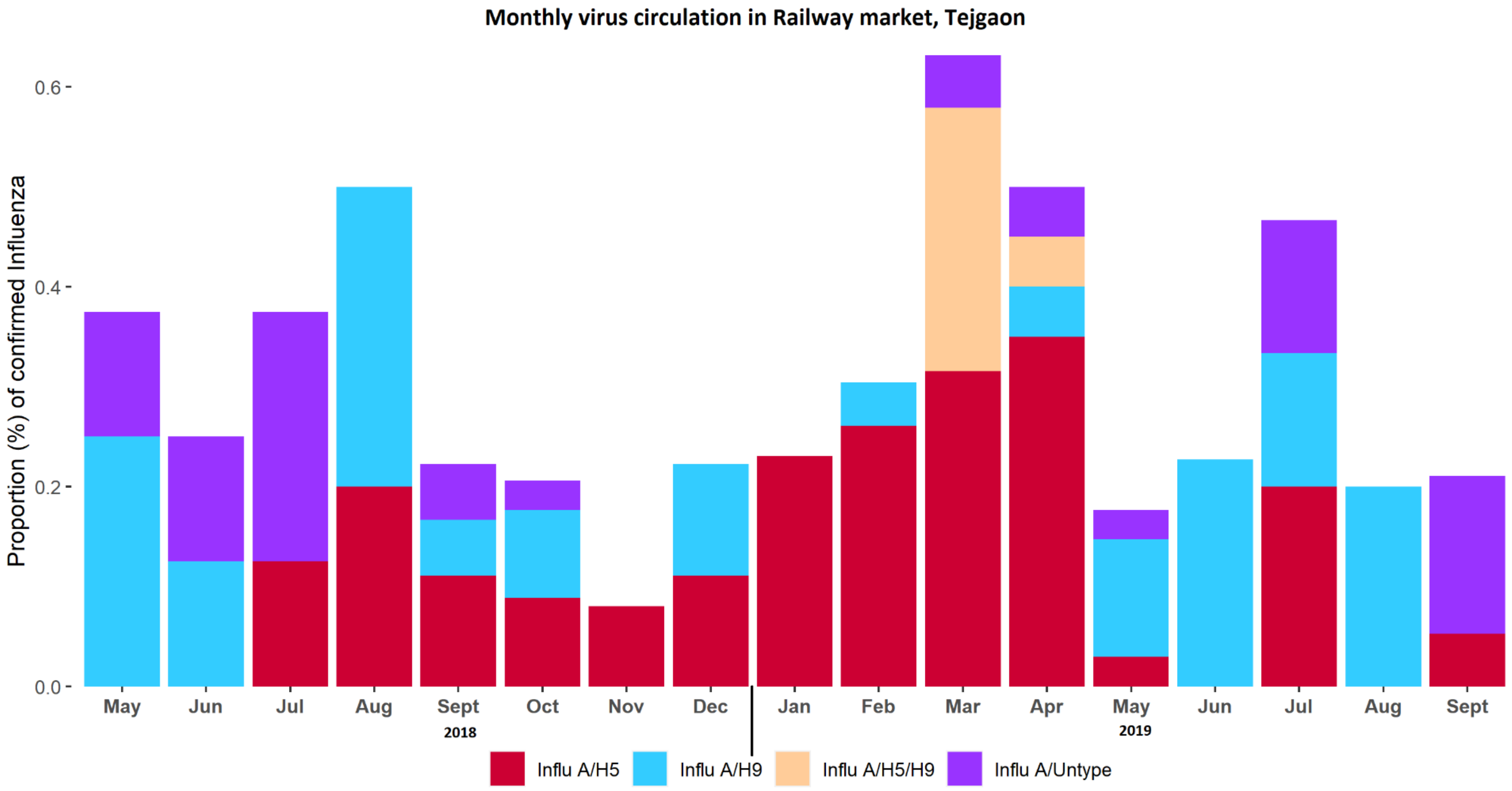

Figure S4: Monthly Variation in the proportion of AIV positive cases

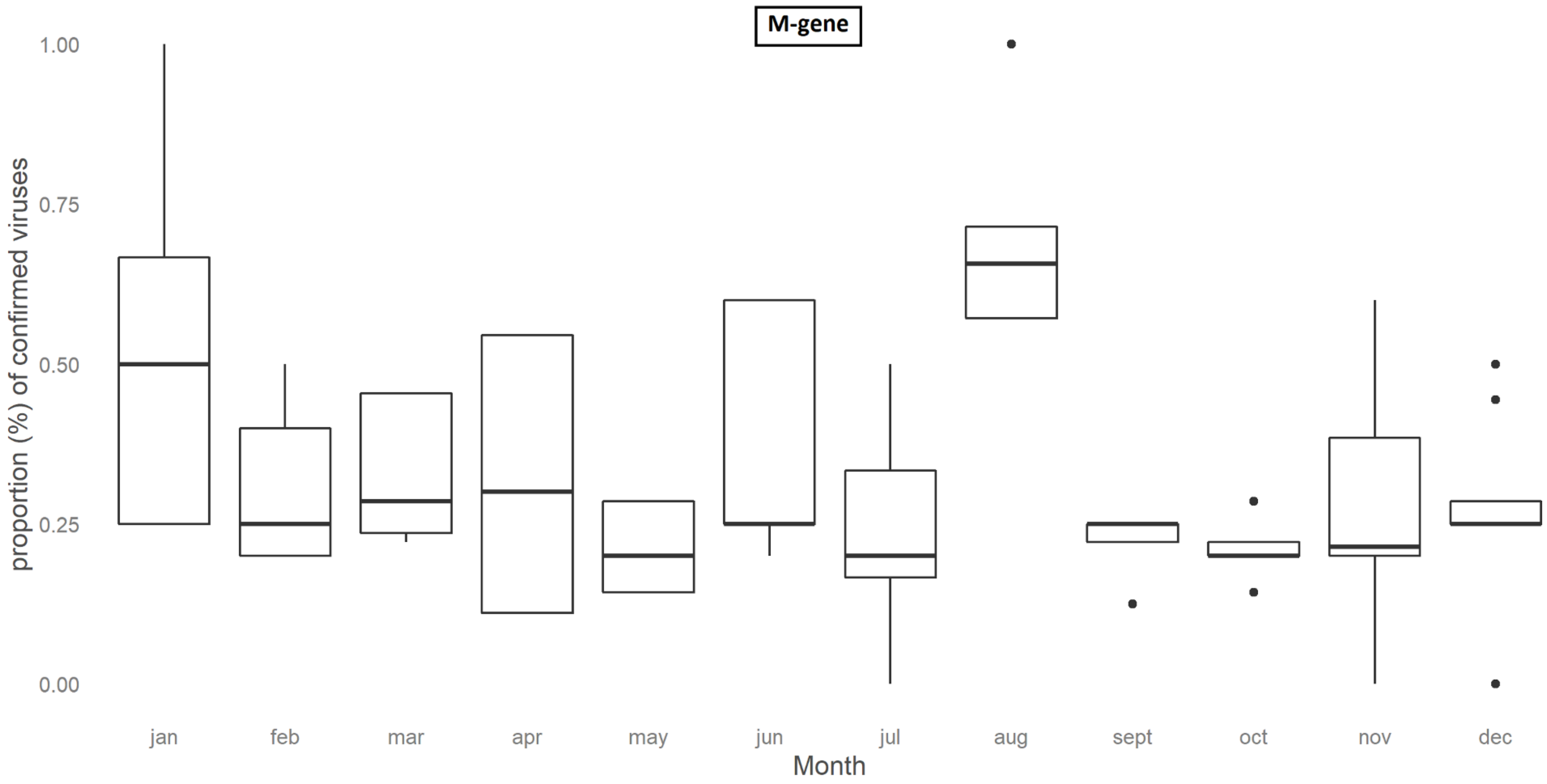

Figure S5: Monthly Variation in the proportion of A/H5 positive cases

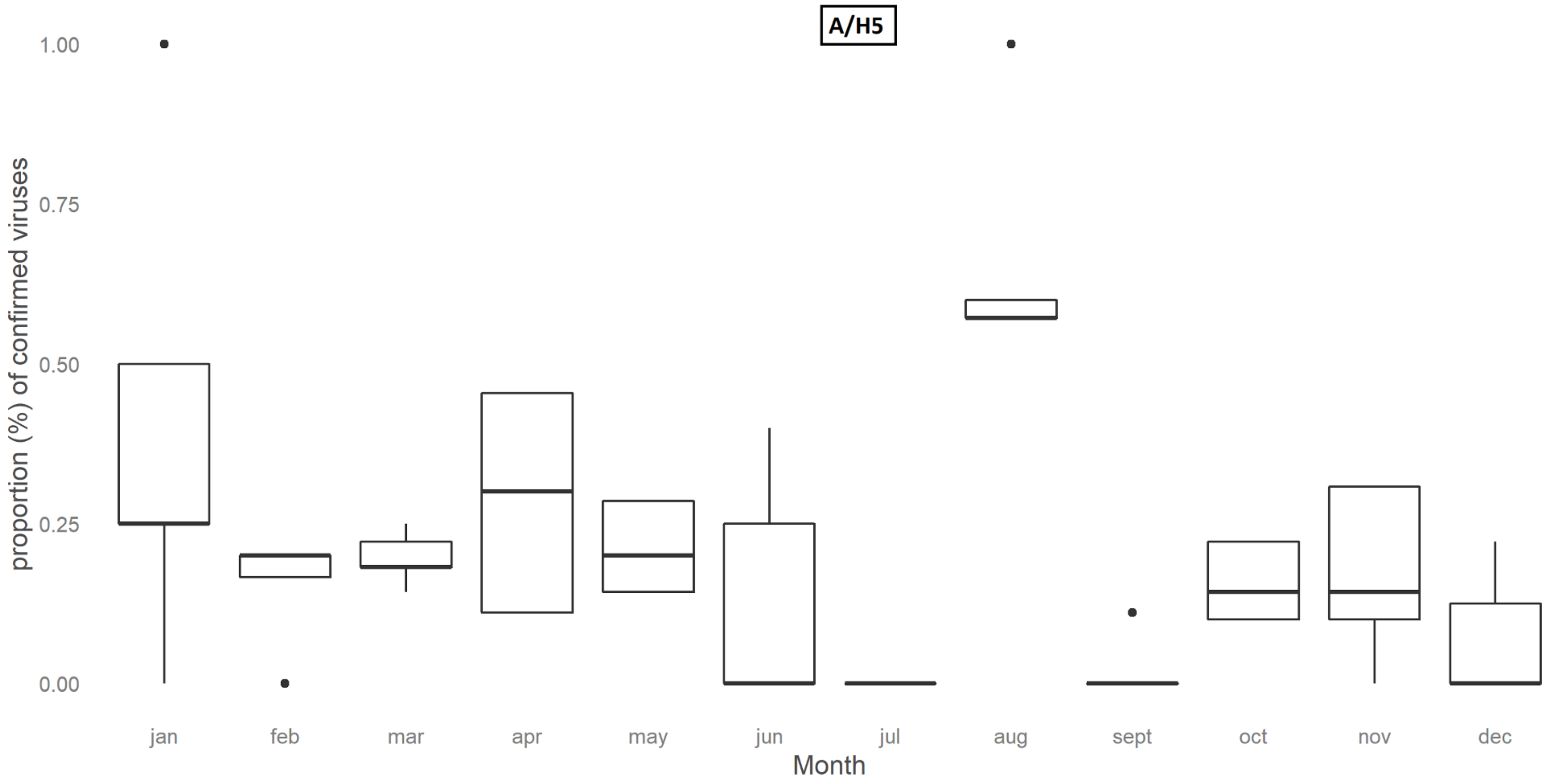

Figure S6: Monthly Variation in the proportion of A/H9 positive cases

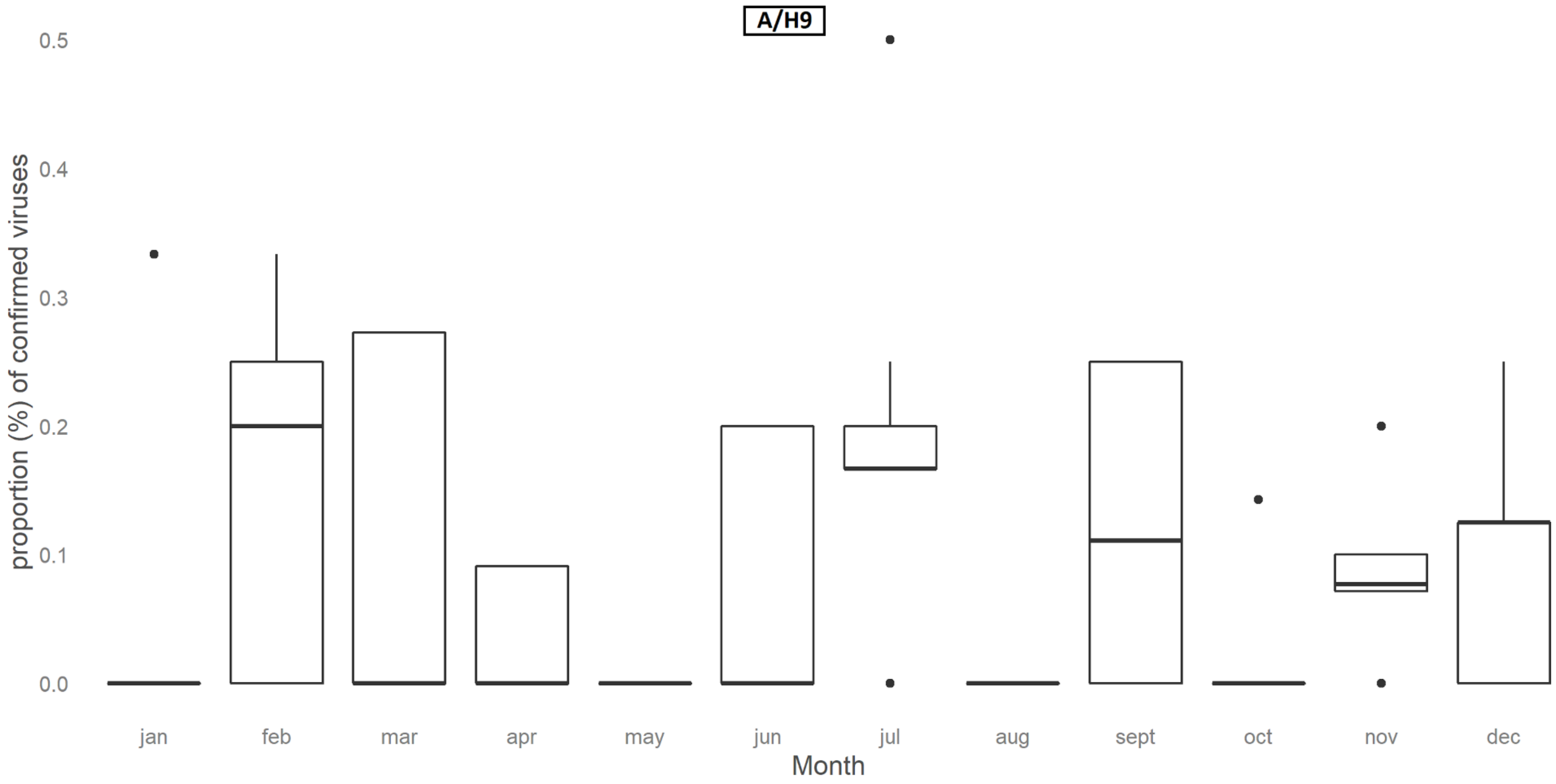

Figure S7: Matrix of values of Cramer's V among the independent variable

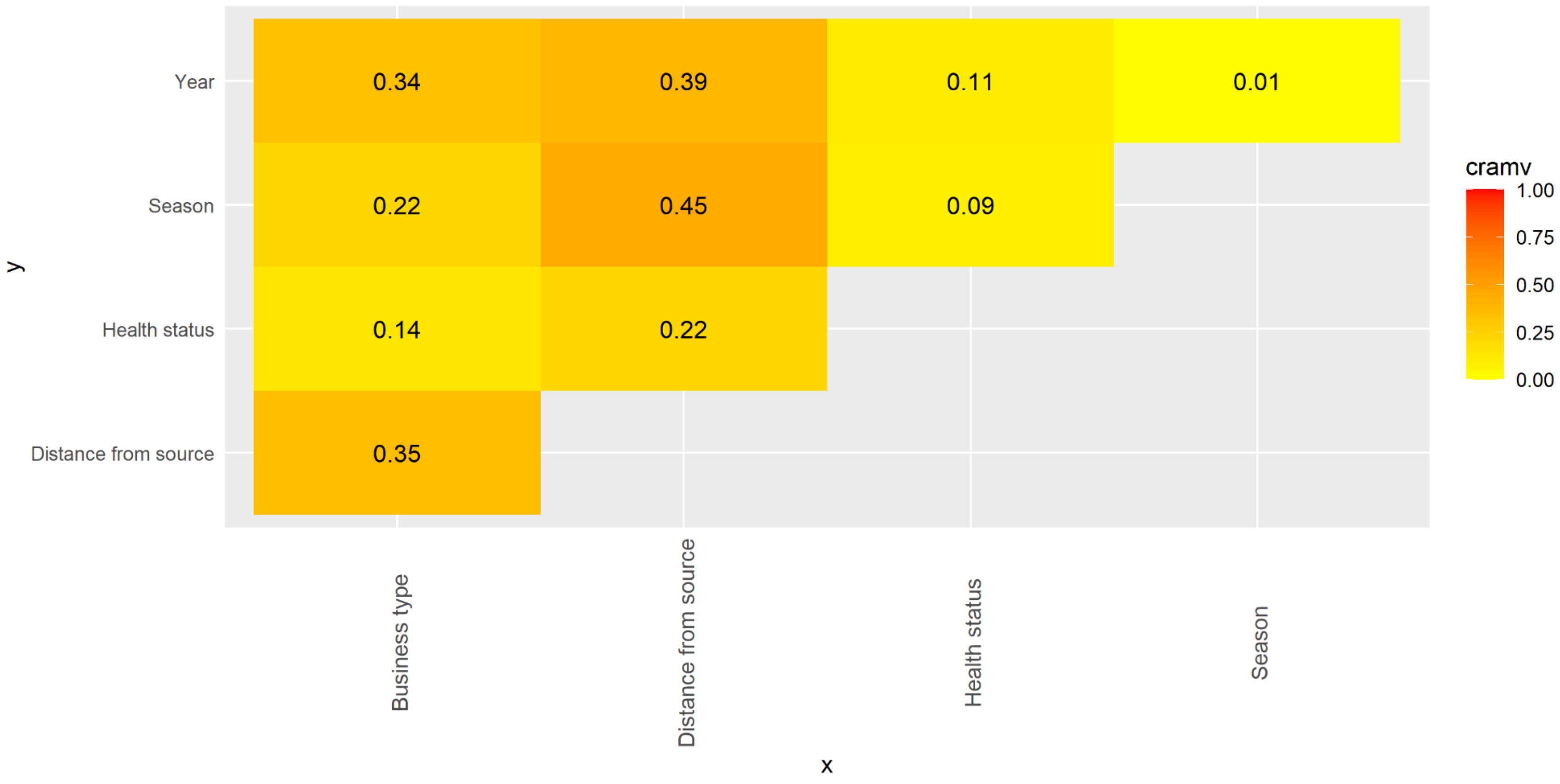

Supplement: Supplementary file 1 [file Data_Sheet_1.pdf]
